# Aggressive Aneurysmal Bone Cyst of the Mandible: A Rare Case of Rapid Expansion and Surgical Management

**DOI:** 10.1002/ccr3.71479

**Published:** 2025-11-16

**Authors:** Fatemeh Mashhadiabbas, Sanaz Gholami Toghchi, Sara Alehossein, Hoorisa Norouzi, Mohammadreza Kashefi Baher

**Affiliations:** ^1^ Department of Pathology Shahid Beheshti University of Medical Sciences Tehran Iran; ^2^ School of Dentistry Shahid Beheshti University of Medical Sciences Tehran Iran; ^3^ Health Research Center, Chamran Hospital Tehran Iran

**Keywords:** aneurysmal bone cysts, bone cyst, jaw cysts, pseudocyst

## Abstract

An aggressive aneurysmal bone cyst of the mandible mimicked malignancy. Diagnosis via biopsy, followed by surgical excision and 2 years of recurrence‐free follow‐up, highlights the importance of diagnosis, treatment, and long‐term monitoring.

## 1 Introduction

Aneurysmal bone cyst (ABC) is a benign, expansile, intraosseous lesion characterized by blood‐filled, osteolytic spaces [1, 2]. Despite being termed a “cyst,” ABC is considered a pseudocyst, as it lacks an epithelial lining on histopathological examination [3]. Initially, ABC was classified as a benign osteoclastic tumor characterized by the presence of giant cells, according to the 2020 WHO Classification of Tumors [4]. In this context, the term “benign central giant cell tumor” has been used to describe certain presentations of aneurysmal bone cysts [5]. However, in the recent WHO classification of odontogenic and maxillofacial bone tumors, ABC is placed under the category of “Giant Cell Lesions and Bone Cysts” [6].

The etiology of ABC remains incompletely understood. Based on early studies, vascular disturbances and traumatic events have both been proposed as potential contributing factors [7, 8]. However, recent investigations have identified a distinction between primary and secondary ABC based on specific genetic alterations, with *USP6* gene rearrangements observed in approximately two‐thirds of cases diagnosed as primary ABC [9]. Moreover, in primary ABC, *USP6* fusions with various genes, such as *CDH11, CNBP, and COL1A1*, have been reported [10]. In contrast, secondary ABC arises from pre‐existing pathological lesions, including giant cell tumors and telangiectatic osteosarcoma, and typically lacks *USP6* gene alterations [7].

The most commonly affected sites are the metaphysis of long bones (67%), the posterior elements of the spine (15%), and the pelvis (9%) [11]. In contrast, involvement of the jaws is rare, accounting for approximately 2% of all ABC cases. When the jaws are affected, the lesion most frequently involves the posterior (molar) region of the mandible [2, 12]. However, occurrence in the anterior region of the mandible is reported to be extremely rare, with only a limited number of cases documented in the literature [13, 14]. Furthermore, cases have also been reported in other craniofacial bones, including the orbit, skull base, and even the sinonasal region [5, 15, 16].

Regarding demographic characteristics, ABC predominantly affects individuals under the age of 30 and shows a female predilection [17].

Several rare variants of ABC have been reported, including giant ABCs [18], multiple metachronous lesions [19, 20], and associated concomitant cemento‐osseous dysplasia (COD) [21, 22].

The clinical and radiographic features of jaw ABC can be highly variable. Clinically, it may present as a rapidly progressive lesion or as a small, asymptomatic growth. The most common clinical finding is swelling, which may occur with or without pain. Less common findings include malocclusion, tooth mobility, and tooth migration [23].

Radiographically, a multilocular or unilocular radiolucency with a typically corticated border is often observed; however, ill‐defined borders may also be present [23]. Moreover, the affected bone may exhibit a “blow‐out” appearance [24].

Histopathologically, it is characterized by multiple blood‐filled cystic spaces lacking epithelial or endothelial lining, surrounded by a narrow layer of reactive bone [3, 7]. Biopsy is essential for establishing a definitive diagnosis and ruling out other possible differential diagnoses.

Various treatment approaches can be considered, including curettage, surgical resection, and sequential instillations of polidocanol [7, 23]. The risk of recurrence after complete treatment is very low. However, it may occur in aggressive lesions or cases of incomplete lesion removal [25]. Moreover, the long‐term prognosis is favorable, and the risk of malignant transformation is extremely low [26].

This study aimed to report a rare case of ABC in an unusual location, with aggressive behavior and rapid growth.

## 2 Case History

**FIGURE 1:**
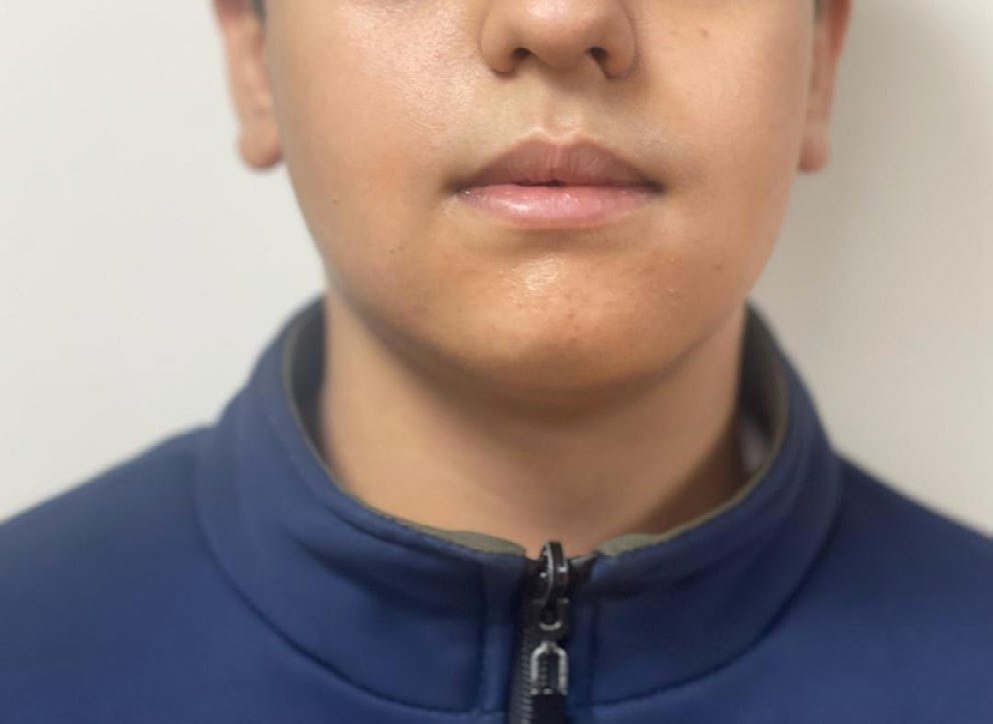
Preoperative clinical image showing swelling in the anterior part of the mandible which proceeds to the mental area and inferior border of the mandible.

A 12‐year‐old boy was referred to the Department of Oral and Maxillofacial Surgery at Shahid Beheshti University of Medical Sciences with a chief complaint of progressive intraoral swelling in the anterior region of the lower jaw. According to his parents, the lesion was first noticed 1 month prior to referral and had progressively increased in size up to the time of presentation. However, no complaints of pain were reported. Extraoral examination revealed mild facial asymmetry due to swelling on the left side of the face (see Figure 1). Intraoral evaluation identified a bony hard lesion extending from the mental region to the inferior border of the mandible.

**FIGURE 2:**
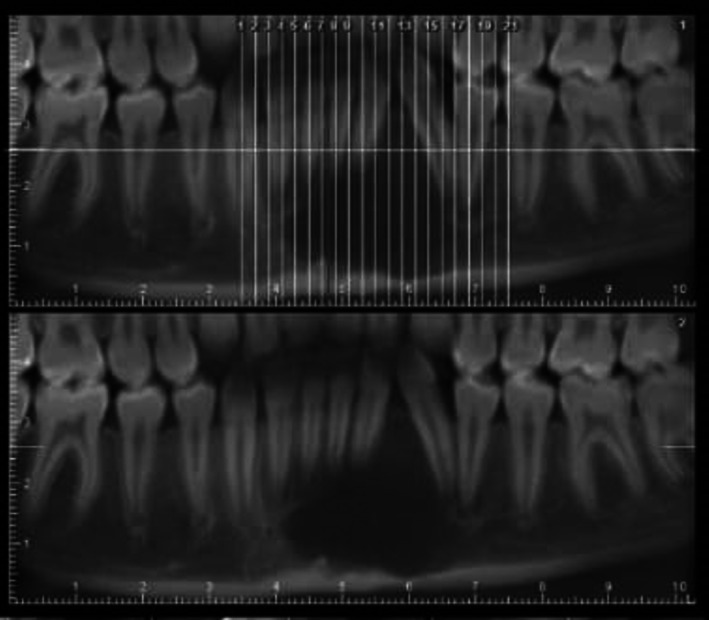
Panoramic radiograph showing a well‐defined multilocular radiolucent lesion in the mandible area of #33–#44 which caused root resorption.

**FIGURE 3:**
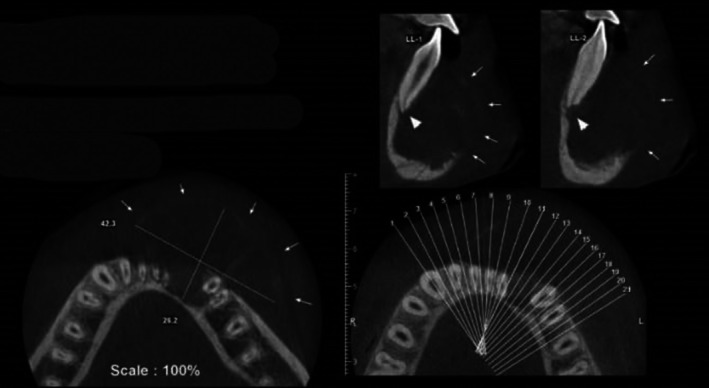
Axial and sagittal CBCT image demonstrating a well‐defined expansile lesion with perforation of the buccal cortex.

Two‐dimensional radiographic examination revealed a well‐defined, multilocular radiolucency with scalloped borders in the mandible. Teeth #33–#44 were affected, showing root resorption and displacement (see Figure 2). Moreover, the lesion invaded the bone and surrounding soft tissue, with labial cortical perforation confirmed by CBCT imaging (see Figure 3).

**FIGURE 4:**
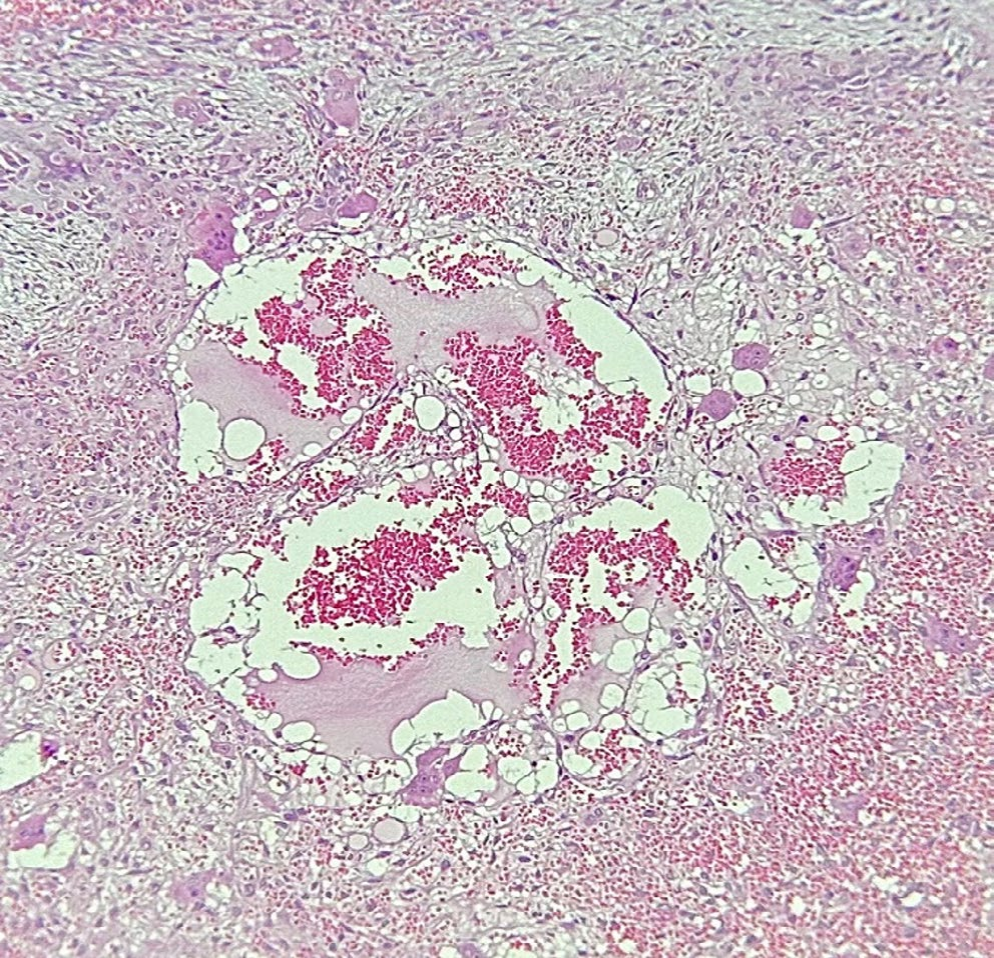
Microscopic image with H&E staining and ×100 magnification showing blood‐filled spaces that are surrounded by numerous multinucleated giant cells.

**FIGURE 5:**
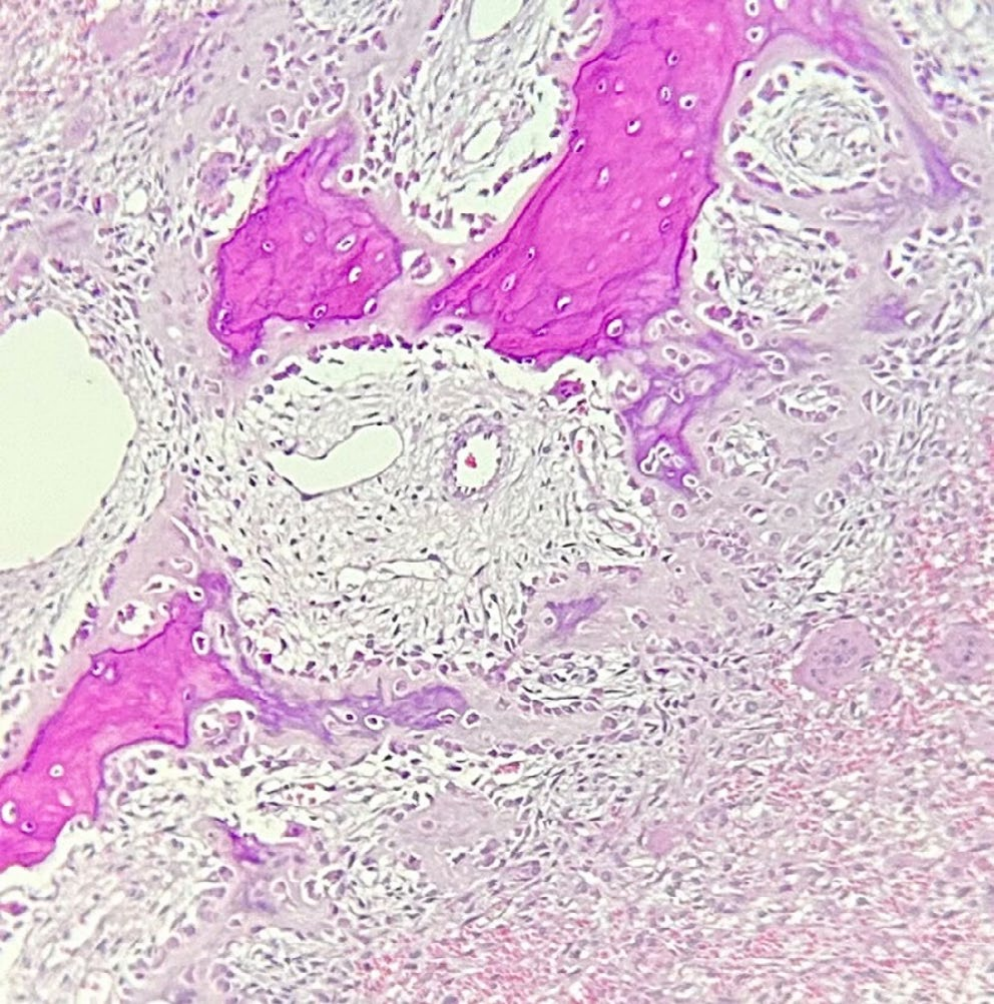
Microscopic image with H&E staining and ×100 magnification showing multiple sinusoidal spaces, multinucleated giant cells, and immature woven bone that are lined by plump osteoblasts.

**FIGURE 6:**
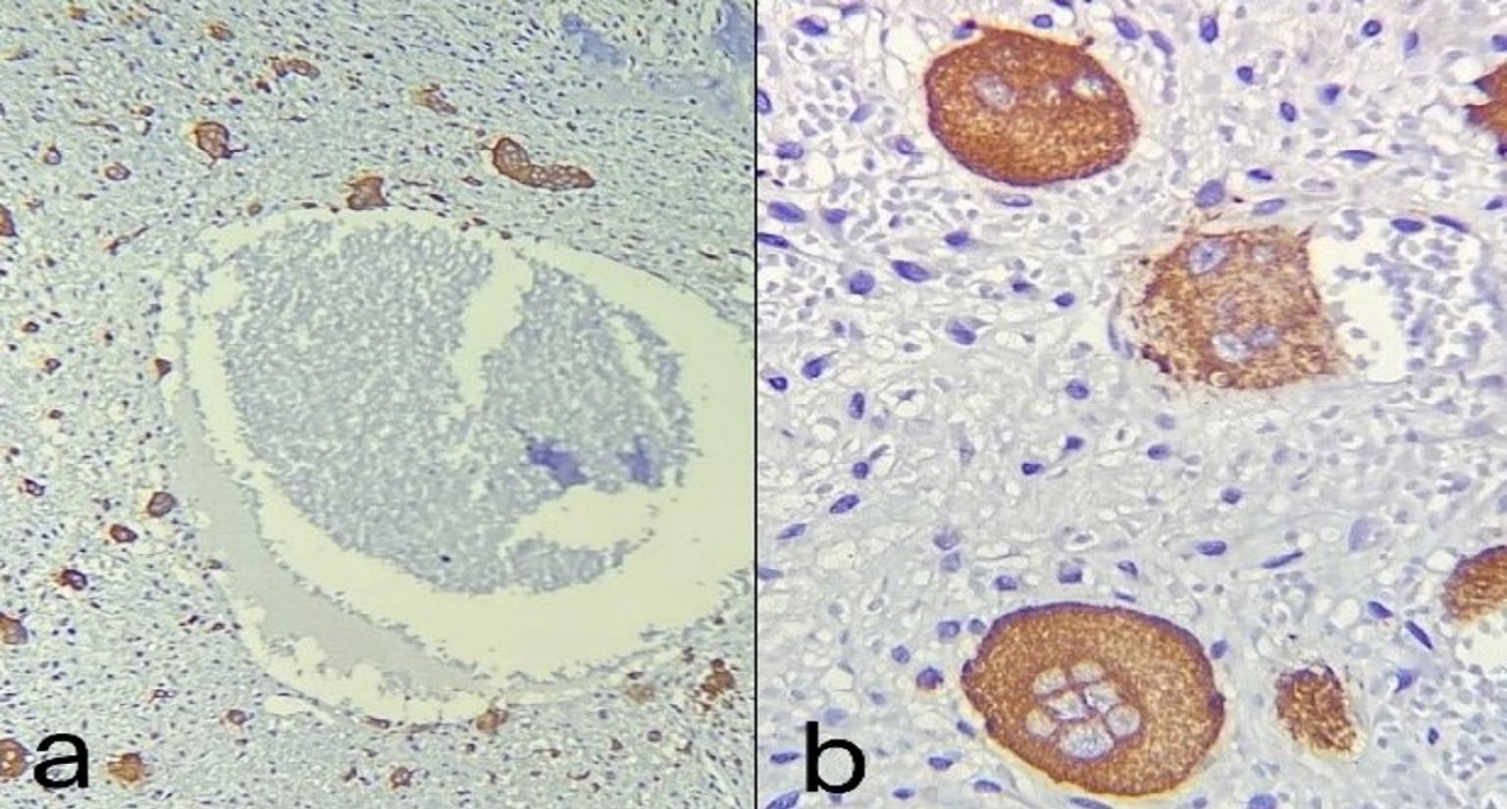
Immunohistochemical staining for CD68 is strongly positive in giant cells. (a): ×100 magnification and (b): ×400 magnification.

The patient initially underwent an incisional biopsy, which confirmed the diagnosis of ABC. Microscopic evaluation revealed numerous sinusoids (pools of red blood cells) of varying sizes lacking endothelial lining, surrounded by loose connective tissue containing multinucleated giant cells and areas of hemorrhage (see Figures 4 and 5). Osteoid and woven bone were identified, indicative of new bone formation. Immunohistochemical (IHC) evaluation demonstrated Ki‐67 positivity in over 20% of cells. Additionally, CD68 staining showed strong positivity in multinucleated giant cells and macrophages (see Figure 6). Overall, based on the clinical, radiographic, histopathologic, and IHC findings, the patient was diagnosed with aggressive ABC.

**FIGURE 7:**
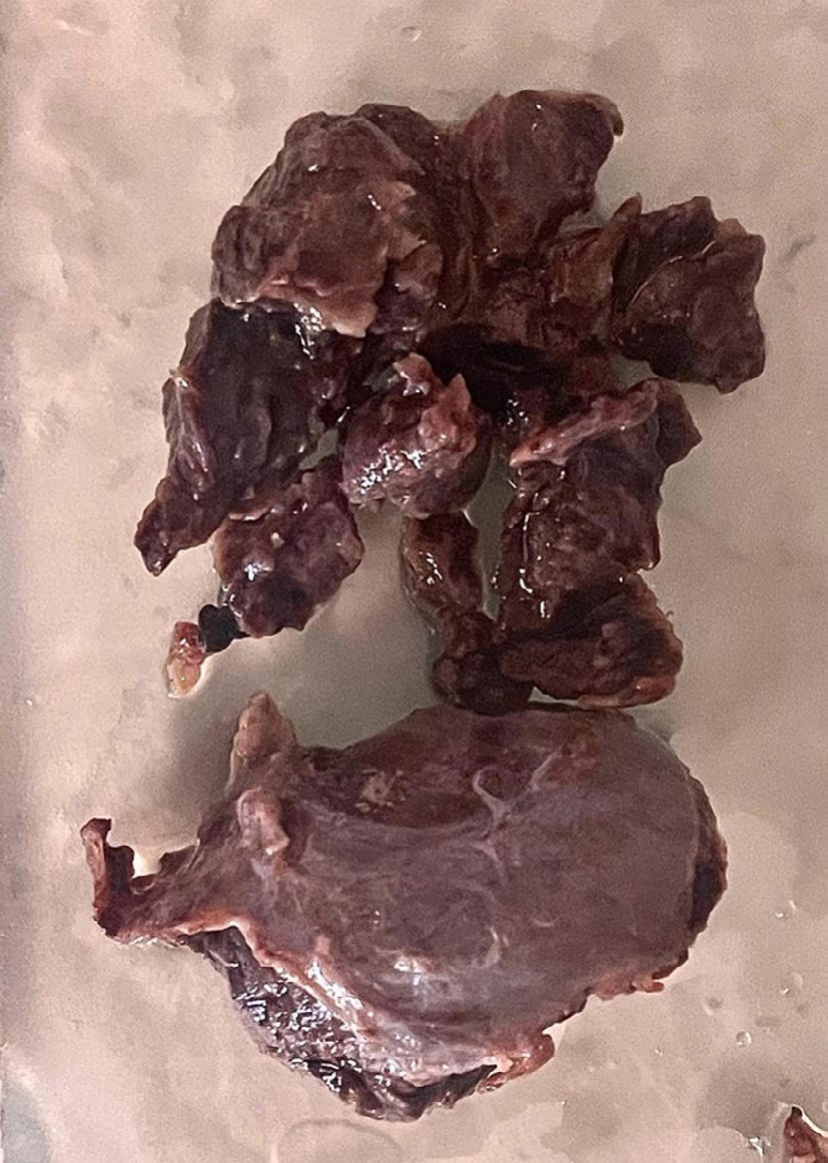
The aneurysmal bone cyst involving the mandible showing multiple blood‐filled spaces.

**FIGURE 8:**
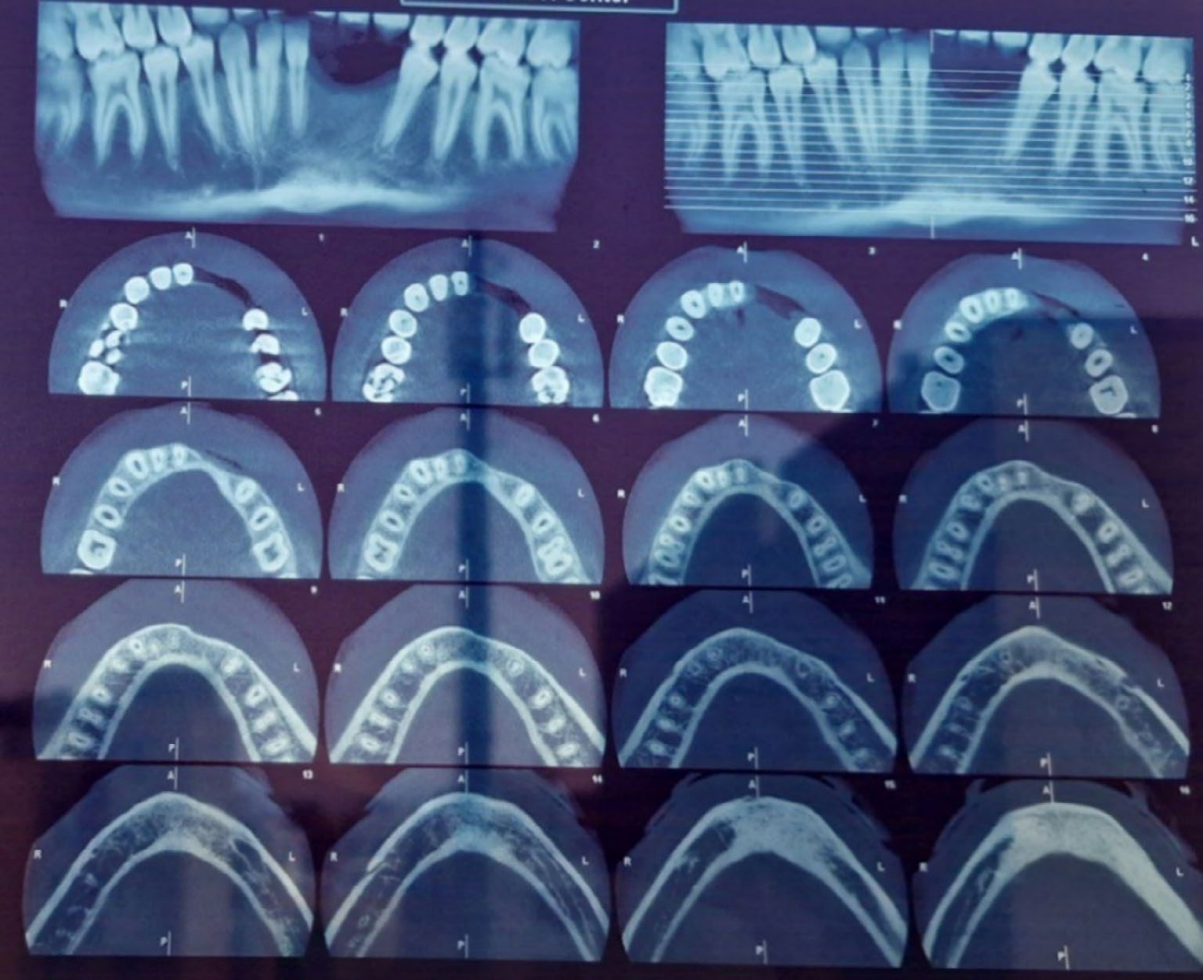
Patient follow‐up after 1 year (Year 2022).

**FIGURE 9:**
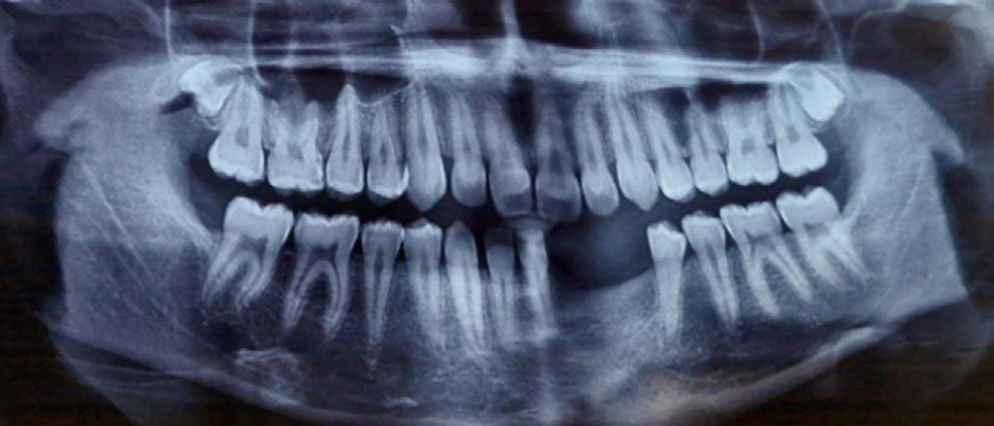
Patient follow‐up after 2 years (Year 2023).

Subsequently, an excisional biopsy was performed, and the lesion was thoroughly curetted (see Figure 7). The surgeon believed that the extent of the lesion suggested a much longer duration, likely developing 2–3 years prior, despite the patient's reported timeline. Two‐year follow‐up examinations revealed no signs of recurrence (see Figures 8 and 9).

Written informed consent was obtained from the patient's parents for publishing his clinical photographs and radiographs in this report according to the journal's patient consent policy.

## 3 Differential Diagnosis, Investigations, and Treatment

Differential diagnoses at the clinical stage included central giant cell tumor, osteosarcoma, and lymphoma. Radiographic differential diagnoses included central giant cell granuloma (the closest radiographic mimic), keratocystic odontogenic tumor, ameloblastic fibroma, ameloblastoma, glandular odontogenic cyst, and telangiectatic osteosarcoma.

Investigations included a detailed patient history obtained from the parents, followed by a comprehensive clinical examination that prioritized extraoral over intraoral assessment. Radiographic evaluation began with conventional two‐dimensional imaging and was followed by cone‐beam computed tomography (CBCT). An incisional biopsy was then performed, and histopathologic examination using H&E staining and IHC confirmed the diagnosis. Treatment involved excisional biopsy of the lesion followed by thorough curettage.

## 4 Conclusion and Results

This case report illustrates a rare instance of aggressive ABC, emphasizing the importance of considering differential diagnoses and the need for an accurate diagnosis based on a combination of clinical, radiographic, and histopathological evaluations. Moreover, arranging long‐term follow‐up after surgical treatment is essential due to the potential, albeit rare, risk of recurrence or malignant transformation.

## 5 Discussion

Aneurysmal bone cyst is defined as a non‐neoplastic, blood‐filled lesion composed of multinucleated giant cells and bony trabeculae [27].

The clinical manifestation of the lesion ranges from slow‐growing, asymptomatic cases to rapidly expanding lesions accompanied by symptoms such as pain [28]. However, in the present case, besides the lesion's rapid growth, aggressive invasion into the bone and surrounding soft tissues was also observed. Moreover, the highly destructive nature of the lesion in this case mimicked malignancy, and the differential diagnoses at the clinical stage included central giant cell tumor, osteosarcoma, and lymphoma. This is consistent with previous case reports, in which the initial clinical diagnosis was malignancy due to the lesion's atypical features [29, 30].

With respect to age, the present case aligns with previous reports; however, regarding the gender, it contrasts with the higher prevalence of this lesion in females [17].

Several studies have reported that certain lesions, termed ABC‐plus lesions, are associated with the occurrence of ABC. These include fibrous dysplasia, central giant cell lesions, chondroblastoma, osteoblastoma, fibromyoma, ossifying fibroma, and chondromyxoid fibroma [12, 17, 31, 32]. Furthermore, ABC is rarely associated with malignant lesions, such as chondrosarcomas and osteosarcomas [33]. It can also be associated with certain medical conditions, including unicameral cysts, hemangiomas, and histiosarcomas [16]. However, in the present case, none of the aforementioned associated lesions were present, and medical investigations revealed no related complications.

The distinguishing feature of this case is the lesion's location. Given the low incidence of jaw ABC and its higher prevalence in the posterior mandible [2, 12], this case is notable for presenting the lesion in the anterior region of the mandible.

The radiographic appearance of ABC can be variable, ranging from radiopaque to radiolucent or mixed radiopaque‐radiolucent lesions. These lesions often exhibit bony expansion or a cystic appearance, sometimes characterized by internal honeycomb or soap bubble patterns [5, 12]. Cortical perforation, as observed in the present case [34], and pathological fracture [5] of the jaw have also been reported. Due to the variable radiographic appearances, several differential diagnoses should be ruled out, including central giant cell granuloma (the closest radiographic mimic), keratocystic odontogenic tumors, ameloblastic fibroma, ameloblastoma, and glandular odontogenic cyst. Moreover, malignancies should also be included in the radiographic as well as clinical differential diagnoses. In this context, telangiectatic osteosarcoma may exhibit a radiographic appearance similar to that of ABC [30]. In the present case, a well‐defined scalloped radiolucency was observed, with characteristic features including labial cortical perforation, root resorption, and tooth displacement.

Histopathological presentation of ABC is typically described as an intraosseous lesion composed of cellular fibrovascular connective tissue containing osteoid, woven bone, and multinucleated giant cells. Moreover, blood‐filled cavities lacking epithelial lining are a hallmark histopathological feature of ABC [35]. Variable amounts of hemosiderin may also be present [5]. The histopathological features of the present case were consistent with the aforementioned characteristics. The lesion's aggressive behavior and nonspecific clinical features raised suspicion for both benign and malignant neoplastic processes in the differential diagnosis. Consequently, IHC staining for the CD68 marker was performed to confirm the presence of multinucleated giant cells and distinguish them from neoplastic cells. Following IHC evaluation and the observation of multiple mitoses, the diagnosis of ABC was confirmed.

The recommended treatment typically involves complete surgical removal of the lesion. However, due to the presence of multiple bony septa dividing the lesion, curettage can sometimes be challenging. Therefore, various treatment approaches are available for different types of ABC, including curettage, surgical excision, cryotherapy, open packing, block resection, and bone grafting [28, 29, 36, 37]. In this case, the lesion was completely removed surgically, followed by curettage. Furthermore, the labial cortical perforation observed on CBCT, was confirmed during surgery.

It is important to note that biopsy is essential for establishing a definitive diagnosis, as giant cell tumors, clinically and radiographically similar to ABC, require a significantly different treatment approach, including postoperative radiation therapy [38]. Misdiagnosis at this stage can compromise the treatment plan and negatively impact patient satisfaction and trust. Although recurrence is rare, it is more likely in aggressive types and in cases of incomplete lesion removal. Therefore, a precise follow‐up program is essential. Accordingly, the patient was enrolled in a scheduled follow‐up program, which revealed no signs of recurrence.

## Author Contributions

**Fatemeh Mashhadiabbas:** conceptualization, data curation, methodology. **Sanaz Gholami Toghchi:** supervision, validation. **Sara Alehossein:** methodology, writing – original draft, writing – review and editing. **Hoorisa Norouzi:** writing – original draft, writing – review and editing.

## Data Availability

The data that support the findings of this study are available from the corresponding author upon reasonable request.
